# Effect of Coronary Calcification Severity on Measurements and Diagnostic Performance of CT-FFR With Computational Fluid Dynamics: Results From CT-FFR CHINA Trial

**DOI:** 10.3389/fcvm.2021.810625

**Published:** 2022-01-03

**Authors:** Na Zhao, Yang Gao, Bo Xu, Weixian Yang, Lei Song, Tao Jiang, Li Xu, Hongjie Hu, Lin Li, Wenqiang Chen, Dumin Li, Feng Zhang, Lijuan Fan, Bin Lu

**Affiliations:** ^1^Department of Radiology, Fuwai Hospital, Chinese Academy of Medical Sciences and Peking Union Medical College, Beijing, China; ^2^Catheterization Laboratories, Fuwai Hospital, Chinese Academy of Medical Sciences and Peking Union Medical College, Beijing, China; ^3^Department of Cardiology, Fuwai Hospital, Chinese Academy of Medical Sciences and Peking Union Medical College, Beijing, China; ^4^Department of Radiology, Beijing Chao-Yang Hospital, Capital Medical University, Beijing, China; ^5^Department of Radiology, Sir Run Run Shaw Hospital, Zhejiang University School of Medicine, Hangzhou, China; ^6^Department of Cardiology, Qilu Hospital of Shandong University, Jinan, China; ^7^Department of Radiology, Qilu Hospital of Shandong University, Jinan, China; ^8^Department of Cardiology, Teda International Cardiovascular Hospital, Tianjin, China; ^9^Department of Radiology, Teda International Cardiovascular Hospital, Tianjin, China

**Keywords:** coronary computed tomography angiography, coronary artery disease, fractional flow reserve, myocardial ischemia, coronary calcification

## Abstract

**Aims:** To explore the effect of coronary calcification severity on the measurements and diagnostic performance of computed tomography-derived fractional flow reserve (FFR; CT-FFR).

**Methods:** This study included 305 patients (348 target vessels) with evaluable coronary calcification (CAC) scores from CT-FFR CHINA clinical trial. The enrolled patients all received coronary CT angiography (CCTA), CT-FFR, and invasive FFR examinations within 7 days. On both per-patient and per-vessel levels, the measured values, accuracy, and diagnostic performance of CT-FFR in identifying hemodynamically significant lesions were analyzed in all CAC score groups (CAC = 0, > 0 to <100, ≥ 100 to <400, and ≥ 400), with FFR as reference standard.

**Results:** In total, the sensitivity, specificity, positive predictive value, negative predictive value, accuracy, and area under receiver operating characteristics curve (AUC) of CT-FFR were 85.8, 88.7, 86.9, 87.8, 87.1%, 0.90 on a per-patient level and 88.3, 89.3, 89.5, 88.2, 88.9%, 0.88 on a per-vessel level, respectively. Absolute difference of CT-FFR and FFR values tended to elevate with increased CAC scores (CAC = 0: 0.09 ± 0.10; CAC > 0 to <100: 0.06 ± 0.06; CAC ≥ 100 to <400: 0.09 ± 0.10; CAC ≥ 400: 0.11 ± 0.13; *p* = 0.246). However, no statistically significant difference was found in patient-based and vessel-based diagnostic performance of CT-FFR among all CAC score groups.

**Conclusion:** This prospective multicenter trial supported CT-FFR as a viable tool in assessing coronary calcified lesions. Although large deviation of CT-FFR has a tendency to correlate with severe calcification, coronary calcification has no significant influence on CT-FFR diagnostic performance using the widely-recognized cut-off value of 0.8.

## Introduction

Fractional flow reserve (FFR) is widely recognized as the gold standard of clinically hemodynamic assessment for patients with coronary artery disease (CAD) (1). While FFR evaluation through invasive procedures is associated with high costs and elevated risk of serious complications. Prior clinical trials have confirmed that FFR assessment based on coronary computed tomography angiography (CT-FFR; CCTA) is an effective non-invasive alternative method to identify ischemia (2–4). One of the most key steps to compute CT-FFR is to construct precise anatomic models of coronary arteries from CCTA, particularly at presence of stenosis (5). However, CCTA is known to have a limited accuracy in stenosis assessment in the vicinity of coronary calcification, due to the beam-hardening artifacts and partial volume effect (6, 7). It is essential to understand the impact of calcification on CT-FFR with regards to accurate identification of coronary lumen boundary. To this end, some prior studies explored the correlation of coronary calcification and discriminatory performance of CT-FFR (8–15). Based on multicenter clinical trial data, CT-FFR showed remarkable improvement in diagnosing ischemia over CCTA and no statistically significant difference in CT-FFR performance was found across calcification severity categories (8, 14, 16). However, the previous studies focused more on the efficacy comparison between CT-FFR and CCTA with increased calcification. The correlation between coronary calcification severity and CT-FFR measurements and diagnostic performance has not been systematically investigated. It is also poorly understood whether and how previous results generalizes to Chinese cohort.

Thus, the objective of current study was to explore the influence of coronary calcification severity on the measurement value and diagnostic performance of CT-FFR by computational fluid dynamics in a prospective multicenter clinical trial carried out in China.

## Methods

### Study Protocol and Patients

This is a sub-study of CT-FFR CHINA trial. CT-FFR CHINA is a multicenter, prospective clinical trial (www.ClinicalTrials.gov; NCT03692936), which aimed to explore the diagnostic performance of CT-FFR over CCTA for identifying flow-limiting lesions, as compared with invasive FFR. It prospectively screened patients with clinically suspected CAD and scheduled invasive coronary angiography (ICA) from 5 clinic sites in China (17). All the patients received CCTA, CT-FFR, clinically indicated ICA, and invasive FFR examinations within 7 days between November 2018, and March 2020. Inclusion criteria were subjects with at least one lesion with 30–90% luminal diameter stenosis in epicardial coronary arteries with diameter ≥ 2.0 mm based on CCTA. Exclusion criteria: (1) previous coronary revascularization, (2) acute coronary syndrome or previous myocardial infarction, (3) heart dysfunction (New York Heart Association class ≥ III), (4) renal dysfunction (glomerular filtration rate <45 ml/kg/1.73m^2^), (5) cardiac artificial device implantation; (6) tachyarrhythmia causing low-quality CCTA images; (7) allergy to iodine contrast media, and contraindications to beta-blockers or nitroglycerin; (8) unevaluable CCTA images for coronary anatomical model construction, (9) pregnancy, (10) operation failure of ICA and FFR. Five local institutional review boards approved the clinical trial protocol. All written informed consents from the included patients were obtained.

**Figure 1:**
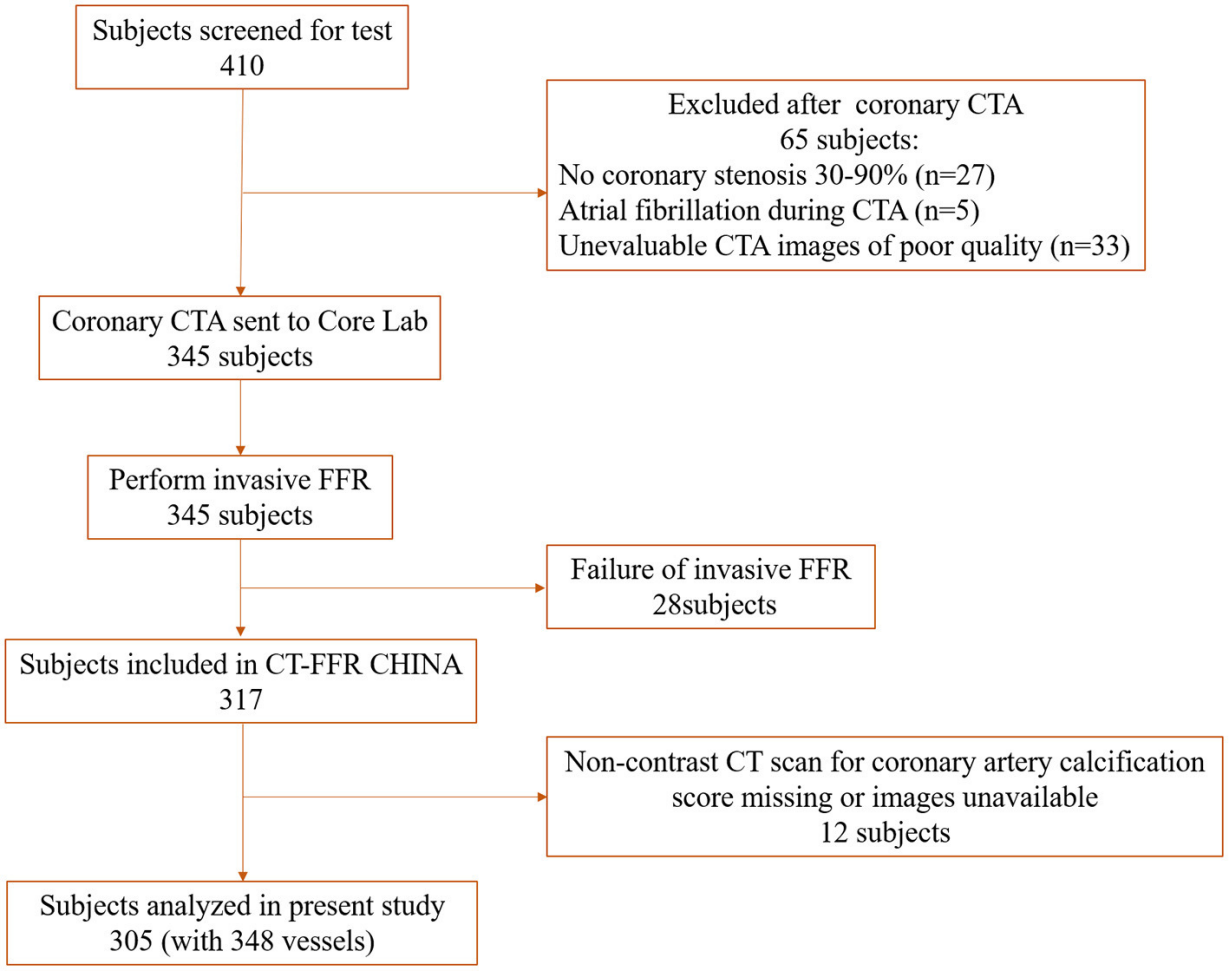
Flow chart of the study. CTA, computed tomography angiography; FFR, fractional flow reserve.

In total, 410 patients were screened. Sixty-five patients were ruled out after CCTA examination: 27 patients with coronary stenosis <30% or >90%, 5 with atrial fibrillation during CTA, and 33 with unevaluable CCTA images. Twenty-eight patients failed to perform FFR operation. Finally, 317 patients were included in the CT-FFR CHINA trial. The flowchart shows in Figure 1.

### CCTA Acquisition and Image Analysis

CCTA was performed using single or dual source CT scanners of ≥ 64 detector rows (Definition Flash/Force, Siemens Healthcare, Forchheim, Siemens; Revolution CT, GE Healthcare, Milwaukee, GE) in 5 clinical centers. CCTA acquisition followed the guidelines of the Society of Cardiovascular Computed Tomography (7). For patients with heart rate >75 beats/min, oral beta-blockers were administered prior to CCTA examination. And sublingual nitroglycerin was used for all patients to induce vessel vasodilation.

Non-enhanced CT images was obtained at 70% R-R interval using a prospective electrocardiographic gating scan (120 KV, slice thickness: 3 mm; no iterative reconstruction was employed). Coronary artery calcification (CAC) scores were calculated by the Agaston method (18). CCTA images was acquired at 35–75% R-R interval with a prospective or retrospective electrocardiogram-triggered technique. The scan parameters were as follows: tube voltage, 70–120 KV depending on the body mass index; tube current, 340 mAs for dual-source CT (Care Dose 4D) and 350–700 mAs for Revolution CT (Smart milliampere); field of view, 20 × 20 cm; reconstruction thickness, 0.625 or 0.75 mm; iterative reconstruction was employed in all machines (Flash: SAFILE; FORCE: ADMIRE; Revolution CT: ASiR).

Luminal stenosis in epicardial coronary arteries with diameter ≥ 2 mm was assessed by a 17-segement coronary model in a blinded manner in CT core laboratories (19). CCTA images were visualized by axial and multiplanar reconstructed images. The diameter stenosis of target vessels was visually categorized into 30–49%, 50–69%, and 70–90% groups. Coronary lesions causing luminal stenosis ≥ 50% was defined as obstructive CAD (20).

### ICA and FFR Measurements

Two cardiologists with more than 10-year experience performed the routine ICA and FFR procedures in line with the Coronary Angiography Guidelines of American Cardiology College (21). To observe and analyze the vessels, more than 2 optimized projection angles were selected for each major coronary artery. Intravenous adenosine (140–180 ug/kg/min) was administrated to achieve hyperemia before FFR evaluation. Then a sensor-tipped pressure guidewire (St Jude Medical, Minneapolis, Minn) was advanced 2–3 cm distal to the stenosis. The location of sensor was recorded to ensure CT-FFR values obtained at the same position. Slowly withdraw the guidewire and FFR value was automatically displayed on the monitor. FFR ≤ 0.80 was considered as hemodynamically significant (22).

### CT-FFR Calculation

CT-FFR calculation software system with computational fluid dynamic principle was developed and provided by Beijing Heartcentury co., Ltd. CT-FFR calculation was performed by core laboratory investigators in a blinded manner, according to the following steps: (1) establishment of 3D coronary anatomical models simulating maximal hyperemia; (2) definition of luminal centerline and boundary; (3) CT-FFR calculation. CT-FFR values of target vessels were obtained at the position recorded during FFR evaluation procedure. CT-FFR ≤ 0.80 was identified as flow-limiting lesions (2, 22).

### Statistical Analysis

MedCalc version 18.2, (MedCalc Software) and SPSS version 26.0 (IBM SPSS Statistics) were used for statistical analysis. Continuous data was expressed as mean ± SD or the median and interquartile ranges. Categorical variables were expressed as frequency (percentages). Spearman's correlation and Bland-Altman methods were used to test the correlation and consistency of invasive FFR and CT-FFR. Vessel-based and patient-based diagnostic accuracy and diagnostic performance (including sensitivity, specificity, positive predictive value [PPV], and negative predictive value [NPV]) of CT-FFR in diagnosing hemodynamically significant lesions were calculated and assessed in all CAC score groups (CAC 0, > 0 to <100, ≥ 100 to <400, and ≥ 400), as compared with invasive FFR. Chi-square test was used to compare accuracy, sensitivity, specificity and PPV, NPV. Receiver operating characteristics (ROC) curve analysis of CT-FFR in detecting ischemia was also performed and compared by DeLong et al. method (23). *P* < 0.05 was considered as statistically significant.

## Results

### Baseline Characteristics of Target Vessels and Patients

Out of 317 patients enrolled in CT-FFR CHINA trial, 305 patients (mean age: 59.2 ± 9.7 years old) and 348 target vessels with evaluable coronary calcification score were included in the current study. The baseline characteristics of included patients and target vessels were shown in Table 1. The median coronary calcium scores were 87.0 (range: 0–2895.0) on per-patient level and 41.0 (range: 0–2012.6) on per-target-vessel level, respectively.

**Table 1:** Baseline characteristics of patient and vessels.

**Patients**	***N* = 305**
Age (years)	59.2 ± 9.7
Sex (Male/Female)	210/95
Diabetes (%)	93 (30.5%)
Hypertension (%)	187 (61.3%)
Hyperlipidemia (%)	198 (64.9%)
Smoking (former/current) (%)	144 (47.2%)
Family history of CAD (%)	41 (12.9%)
Body mass index (kg/m^2^)	25.8 ± 3.3
**Dominance type of coronary artery**
Right dominance/Left dominance/Balance	288/12/5
Patients with obstructive CAD based on CCTA	236 (77.4%)
Patients with FFR ≤ 0.8	155 (51.0%)
CAC scores	87.0 (0, 2895.0)
**Vessels**	***N*** **=** **348**
**Target vessels**
LADs/ RCAs/ LCXs	237/59/52
**Vessel's diameter stenosis by CCTA**
30–49%/50–69%/70–90%	83/133/132
FFR ≤ 0.8	162 (46.6%)
CAC score of target vessels	41.0 (0, 2012.6)

*CAD, coronary artery disease; CCTA, coronary computed tomography angiography; FFR, fractional flow reserve; LAD, left anterior descending coronary artery; RCA, right coronary artery; LCX, left circumflex artery; CAC, coronary artery calcium. Values are n (%) and median(ranges)*.

The mean FFR values of vessels in CAC = 0, CAC > 0 to <100, CAC ≥ 100 to <400, and CAC ≥ 400 groups were decreased in turns (0.81 ± 0.14, 0.78 ± 0.13, 0.78 ± 0.11, and 0.74 ± 0.14; *p* = 0.016) (Table 2). The percentages of vessels with ICA stenosis ≥ 50% were high in CAC = 0 (67.5%) and CAC ≥ 400 (68%) groups, however, with no statistical significance.

**Table 2:** Results of CCTA, ICA, and FFR across CAC score categories in vessels.

	**CAC = 0 (*N* = 77)**	**CAC > 0 to <100 (*N* = 149)**	**CAC ≥ 100 to <400 (*N* = 97)**	**CAC ≥ 400 (*N* = 25)**	***P***
FFR	0.81 ± 0.14	0.78 ± 0.13	0.78 ± 0.11	0.74 ± 0.14	0.016
CT-FFR	0.81 ± 0.15	0.78 ± 0.15	0.75 ± 0.16	0.75 ± 0.15	0.008
FFR ≤ 0.8 (%)	27 (35.1)	73 (49.0)	47 (48.5)	15 (60.0)	0.094
ICA ≥ 50% (%)	52 (67.5)	94 (63.1)	58 (59.8)	17 (68.0)	0.719
CCTA ≥ 50% (%)	59 (76.6)	109 (73.2)	72 (74.2)	25 (100)	0.032

*ICA, invasive coronary angiography; CT-FFR, computed tomography derived fractional flow reserve; CCTA, coronary computed tomography angiography; FFR, fractional flow reserve; CAC, coronary artery calcium. Values are or n (%). FFR: mean ± SD*.

### Correlation and Consistency of CT-FFR Measurements and Invasive FFR Values

**Figure 2:**
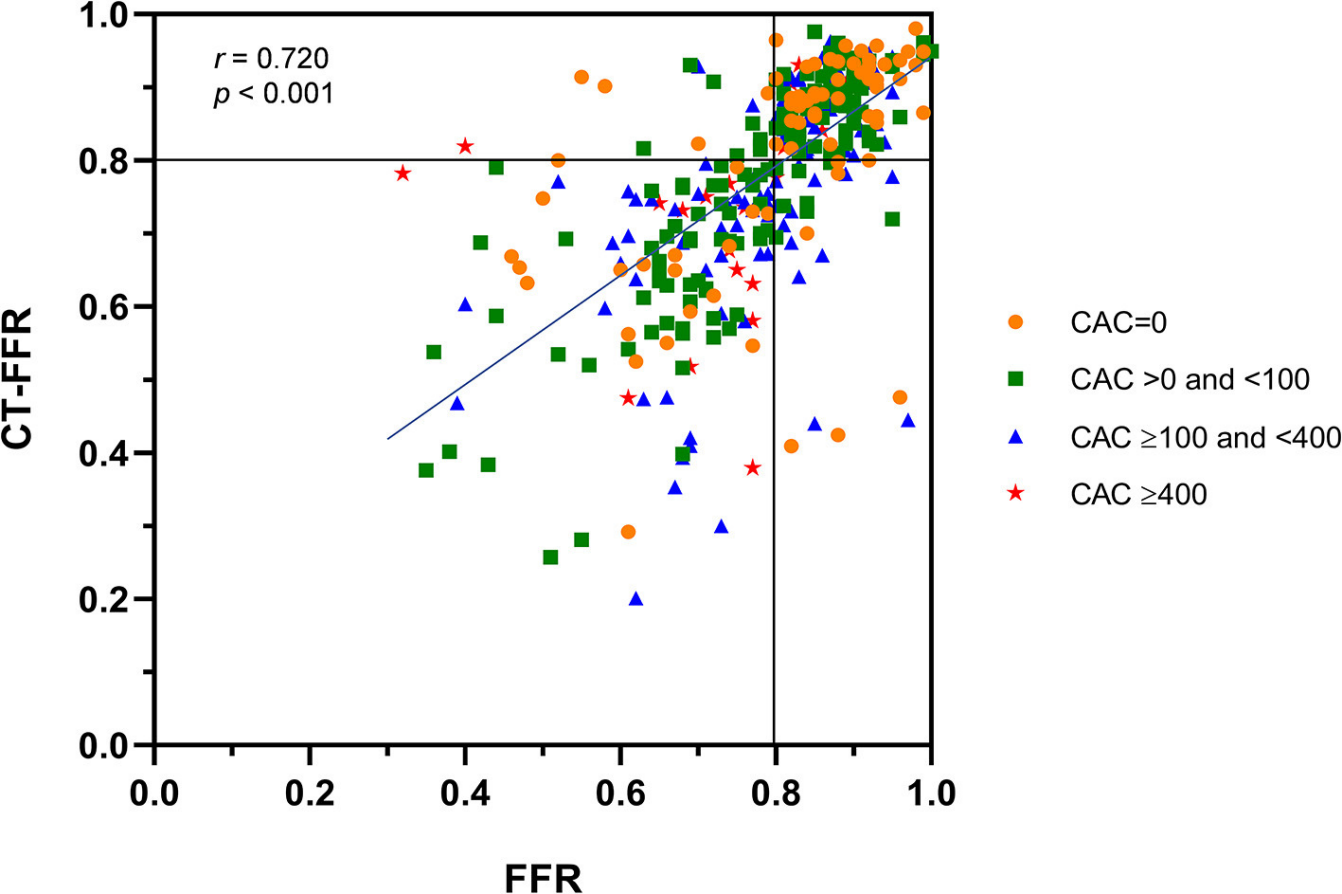
Correlation of CT-FFR to Invasive FFR. Good correlation of CT-FFR measurements to invasive FFR values is observed on the per-vessel level (Spearman's correlation coefficient = 0.720, *p* < 0.001). CT-FFR, computed tomography derived fractional flow reserve; FFR, fractional flow reserve.

Spearman's rank correlation analysis showed a good correlation of invasive FFR and CT-FFR on a per-vessel level (*r* = 0.720, *p* < 0.001) (Figure 2). The area of scatter plot can be divided into 4 quadrants by line CT-FFR = 0.8 and line FFR = 0.8. Most of the dots in CAC = 0 group was concentrated in the right upper quadrant and close to dot (0.8, 0.8), but dots with CAC ≥ 400 was mostly scattered in the left lower quadrant and far from (0.8, 0.8). The rest dots representing vessels with CAC > 0 to <100 and CAC ≥ 100 to <400 distributed relatively balanced in the right upper and left lower quadrants.

**Figure 3:**
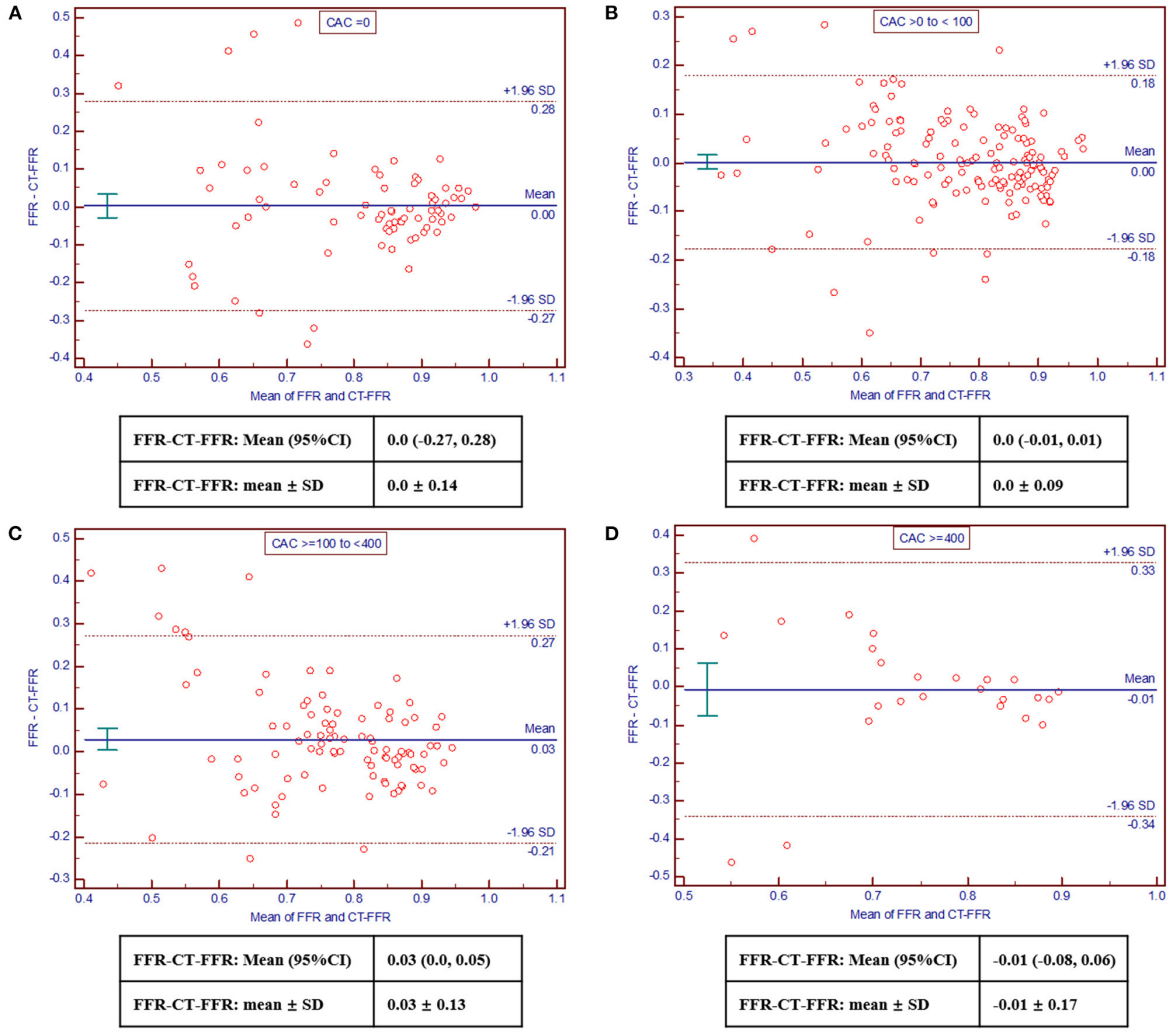
Bland-Altman plots comparing CT-FFR with invasive FFR. Good and acceptable consistency of CT-FFR and invasive FFR across CAC score categories was observed [**(A)**, CAC = 0; **(B)**, CAC > 0– <100; **(C)**, CAC ≥ 100– <400; **(D)**, CAC ≥ 400]. CAC, coronary artery calcium; CT-FFR, computed tomography derived fractional flow reserve; FFR, fractional flow reserve.

The Bland-Altman analysis revealed good consistency of FFR and CT-FFR values across all CAC score categories, which was acceptable (Figure 3). And the bias was as following: −0.01 (95% limits of agreement: −0.03 to 0.03) for CAC = 0, 0.01 (−0.01 to 0.01) for CAC > 0 to <100, 0.03 (0.01–0.06) for CAC ≥ 100 to <400, 0.0 (−0.08–0.06) for CAC ≥ 400, respectively. Besides, the absolute difference of CT-FFR and FFR tended to elevate with increased CAC scores (CAC = 0: 0.09 ± 0.10; CAC > 0 to <100: 0.06 ± 0.06; CAC ≥ 100 to <400: 0.09 ± 0.10; CAC ≥ 400: 0.11± 0.13; *p* = 0.246), although no statistically significant difference was observed.

### Patient-Based and Vessel-Based Diagnostic Performance and Accuracy of CT-FFR Across Calcification Severity (CAC) Categories

In total, the sensitivity, specificity, PPV, NPV, accuracy, and area under receiver operating characteristics curve (AUC) of CT-FFR for identifying ischemia were 85.8% (79.5–90.8%), 88.7% (83.3–92.9%), 86.9% (81.5–90.9%), 87.8% (83.0–91.3%), 87.1% (83.7–90.4%), 0.90 (0.86–0.93) on the per-patient level and 88.3% (82.3–93.0%), 89.3% (83.3–93.8%), 89.5% (84.3–93.2%), 88.2% (82.8–92.0%), 88.9% (84.9–92.2%), 0.88 (0.84–0.92) on the per-vessel level, respectively.

**Figure 4:**
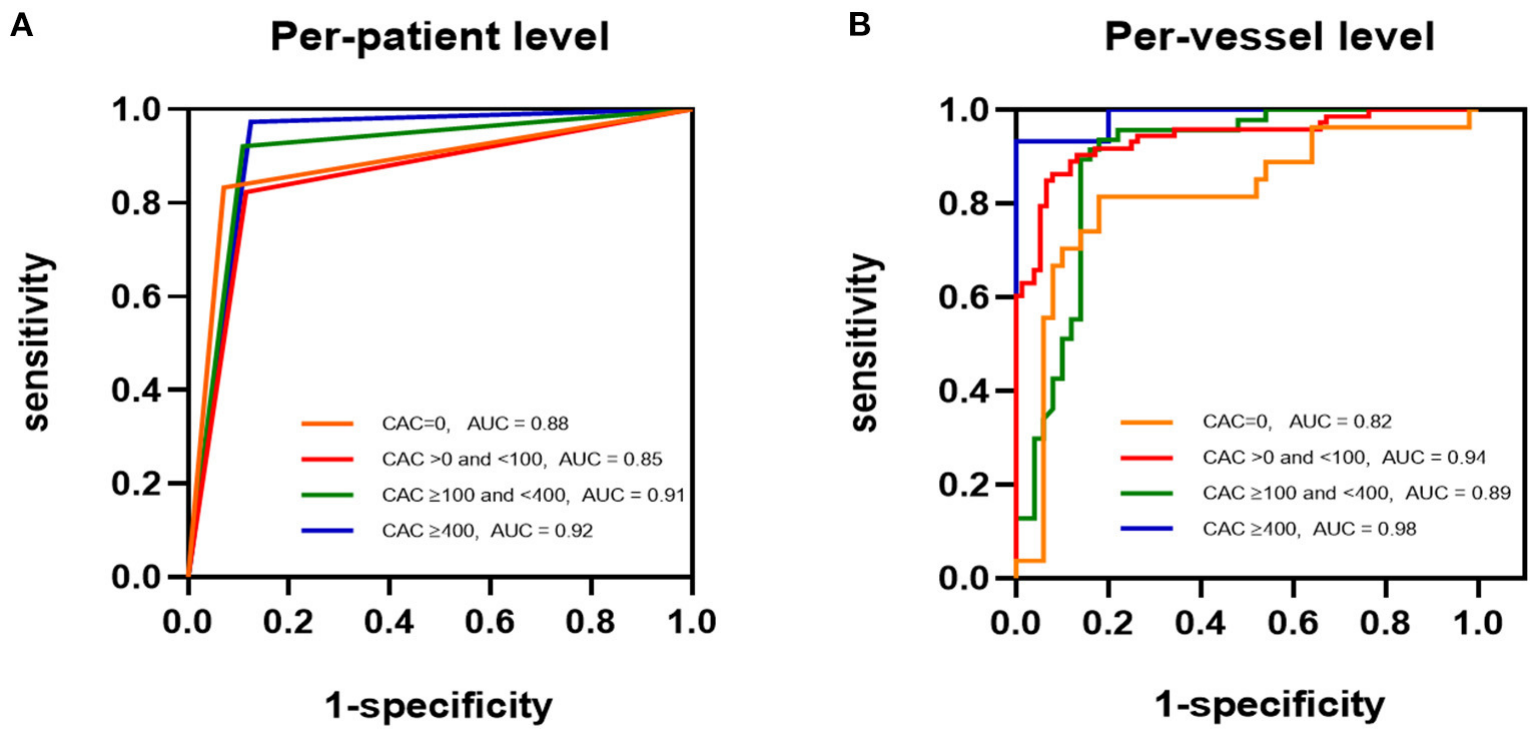
ROCs of CT-FFR Identifying Flow-limiting Lesions. ROCs for detection of ischemia with CT-FFR among all CAC score groups [**(A)**, per-patient level; **(B)**, per-vessel level]. CAC, coronary artery calcium; CT-FFR, computed tomography derived fractional flow reserve.

A total of 348 target vessels were divided into CAC = 0 (77/348, 22.1%), > 0 to <100 (149/348, 42.8%), ≥ 100 to <400 (97/348, 28.9%), and ≥ 400 (25/348, 7.2%) groups. The vessel-based sensitivity, specificity, NPV, and accuracy of CT-FFR were not significantly different among all CAC score categories (Table 3). CAC = 0 and CAC ≥ 400 groups possessed the lowest and highest PPV values, respectively (CAC = 0: 71.0% [56.8%−81.9%]; CAC > 0 to <100: 91.3% [82.9%−85.8%]; CAC ≥ 100 to <400: 83.0% [72.9%−89.9%]; CAC ≥ 400: 100%; *p* = 0.043). Meanwhile, ROC curve analysis revealed the poorest diagnostic power of CT-FFR in CAC = 0 group and highest power in CAC ≥ 400 group (CAC = 0: AUC = 0.82 [95% CI: 0.71–0.89]; CAC > 0 to <100: AUC = 0.94 [95% CI: 0.89–0.97]; CAC ≥ 100 to <400: AUC = 0.89 [95% CI: 0.81–0.94]; CAC ≥ 400: AUC = 0.98 [95% CI: 0.84–1.00]) (Figure 4, Table 3). However, no statistically significant difference was observed. It was noticed that fewest cases were classified into CAC ≥ 400 group.

**Table 3:** Diagnostic performance and accuracy of CT-FFR across CACs categories on per-patient and per-vessel level.

	***N***	**CAC score**	**Accuracy**	**Sensitivity**	**Specificity**	**PPV**	**NPV**	**AUC**
**Per-patient level**
0	46	0	89.1 (78.3–97.8)	83.33 (58.6–96.4)	92.86 (76.5–99.1)	88.2 (66.0–96.7)	89.7 (75.4–96.1)	0.88 (0.75–0.96)
> 0 to <100	114	36.35 (1–99)	85.1 (78.1–91.2)	82.26 (70.5–90.8)	88.46 (76.6–95.6)	89.5 (79.9–94.8)	80.7 (70.8–87.8)	0.85 (0.78–0.91)
≥ 100 to <400	84	216 (100–395)	90.5 (83.3–96.4)	92.11 (78.6–98.3)	87.13 (76.4–96.4)	87.5 (75.3–94.2)	93.2 (82.1–97.6)	0.91 (0.82–0.96)
≥ 400	61	630.5 (423.2–2895.0)	93.4 (86.9–98.4)	97.30 (85.8–99.9)	87.50 (67.6–97.3)	92.3 (80.6–97.2)	95.5 (75.1–99.3)	0.92 (0.83–0.98)
*P*	–	–	0.368	0.107	0.902	0.913	0.152	all *p* > 0.0.5
**Per-vessel level**
0	77	0	81.8 (74.0–90.0)	81.48 (61.9–93.7)	82.0 (68.6–91.4)	71.0 (56.8–81.9)	89.1 (78.6–94.8)	0.82 (0.71–0.89)
> 0 to <100	149	29(0.1–99.7)	88.6 (83.2–93.3)	86.3 (76.2–93.2)	92.11 (83.6–97.0)	91.3 (82.9–85.8)	87.5 (79.7–92.6)	0.94 (0.89–0.97)
≥ 100 to <400	97	207.0 (102.5–394.9)	86.6 (79.4–92.8)	93.62 (82.5–98.7)	82.0 (68.6–91.4)	83.0 (72.9–89.9)	93.2 (81.9–97.6)	0.89 (0.81–0.94)
≥ 400	25	713.86 (403.2–2012.6)	96.0 (88.0–100)	93.33 (68.1–99.8)	100.0 (69.2–100.0)	100	90.0 (60.1–98.5)	0.98 (0.84–1.00)
*P*	–	–	0.213	0.089	0.063	0.043	0.769	all *p* > 0.0.5

*CT-FFR, CT derived fractional flow reserve; CAC, coronary artery calcium; PPV, positive predictive value; NPV, negative predictive value; AUC, area of receiver operating characteristic curve. CAC score: median(range). Accuracy, sensitivity, specificity, PPV, NPV: % (95% confidence interval). AUC: value (95% confidence interval)*.

**Figure 5:**
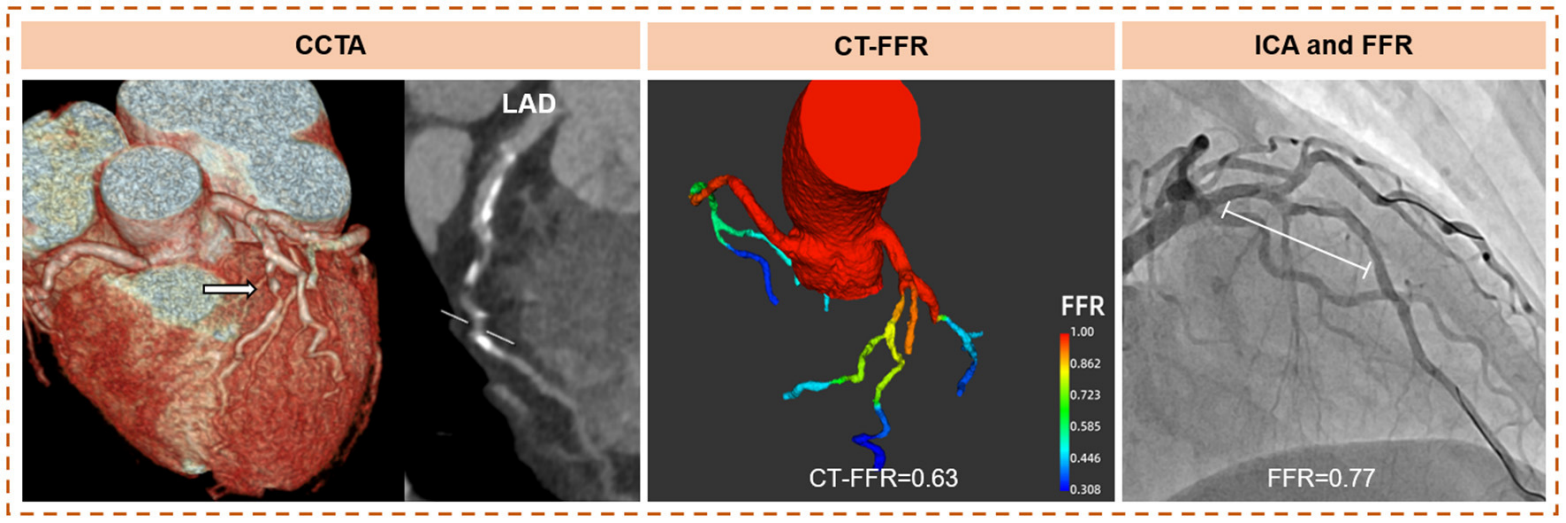
Case of CT-FFR estimating coronary lesions with severe calcification. Example of a 66-year-old female with stable chest pain. The CAC scores of LAD, LCX, and RCA were 599.6, 216.4, and 716.2. CCTA showed multiple calcified and mixed plaques causing more than 70% stenosis in the proximal and mid LAD. CT-FFR demonstrated functional ischemia (CT-FFR = 0.63) caused by LAD stenosis, and confirmed by FFR (FFR = 0.77). The deviation of CT-FFR was 0.14 in the situation of severe coronary calcification. With the widely-accepted threshold of ischemia ( ≤ 0.8), CT-FFR made a correct dichotomous diagnosis. LAD, left anterior descending coronary artery; LCX, left circumflex artery; RCA, right coronary artery; CT-FFR, computed tomography derived fractional flow reserve; FFR, fractional flow reserve; CAC, coronary artery calcium; CTA, computed tomography angiography.

Three hundred and five patients were classified into CAC = 0 (46/305, 15.1%), CAC > 0 to <100 (114/305, 37.4%), CAC ≥ 100 to <400 (84/305, 27.5%), and CAC ≥ 400 (61/305, 20.0%). On a per-patient level, the accuracy, and sensitivity, specificity, PPV, and NPV of CT-FFR were not significantly different across CAC score categories (Table 3). The highest diagnostic power was also observed in CAC ≥ 400 group (CAC = 0: AUC = 0.88 [95%CI: 0.75–0.96]; CAC > 0 to <100: AUC = 0.85 [95% CI: 0.78–0.91]; CAC ≥ 100 to <400: AUC = 0.91 [95%CI: 0.82–0.96]; CAC ≥ 400: AUC = 0.92 [95% CI: 0.83–0.98]), with no statistical significance (Figure 4, Table 3). The case of CT-FFR estimating coronary lesions with severe calcification displays in Figure 5.

## Discussion

This study investigated the impact of coronary calcification severity on the measurements and diagnostic performance of CT-FFR based on computational fluid dynamics algorithm. The results showed absolute difference of CT-FFR measurements and invasive FFR tended to elevate with increased CAC scores (CAC = 0: 0.09 ± 0.10; CAC > 0 to <100: 0.06 ± 0.06; CAC ≥ 100 to <400: 0.09 ± 0.10; CAC ≥ 400: 0.11± 0.13; *p* = 0.246). Although the highest discriminatory power of CT-FFR was showed in CAC ≥ 400 group, no statistically significant difference was observed across all CAC score categories on both per-vessel and per-patient levels.

Coronary calcification is a very challenging issue for CT-FFR calculation. The blooming artifacts and partial volume effect of calcification could shelter the vessel lumen, and often cause biased interpretation of coronary stenosis degree in CCTA images. To our knowledge, the effect of calcification severity on the measurements and discriminatory ability of CT-FFR has not been systematically explored. Our results showed a tendency of larger CT-FFR measurement deviation with increased CAC score. Precise CT-FFR measurements relies on accurate anatomic model of coronary arteries based on good-quality CCTA images (5). The presence of coronary calcification impaired CT-FFR measurements due to the inaccurate coronary model construction based on CCTA, particularly the inadequate recognition of lumen boundary.

However, no statistically significant difference in CT-FFR performance was observed across all CAC score categories on both per-vessel and per-patient levels in our study. This result is similar to the sub-study of NXT trial and MACHINE registry (8, 14). It's still noticed that the AUC values of CT-FFR in identifying ischemia in CAC ≥ 400 category are the highest on both per-vessel and per-patient levels. The possible reason could be related to the flow-limiting degree of lesions and the single cut-off value of 0.8. The progression of coronary atherosclerotic lesions is generally accompanied by increased calcification (24). The baseline analysis of our study showed the severest stenotic grade and lowest mean FFR value (0.74) in vessels with severe calcification (CAC ≥ 400). Meanwhile, FFR ≤ 0.8 is widely recognized as the threshold of ischemia (25). It is a definite value rather than a range. The discrimination of ischemia by CT-FFR is more likely to be correct when the difference between real FFR value and the threshold of ischemia (FFR ≤ 0.8) is large (26). On the contrary, the dichotomous diagnosis is prone to be error when actual FFR is close to 0.8, even with a slight deviation of CT-FFR measurements. Accordingly, the diagnostic performance of CT-FFR in vessels with severe calcification and resultant severe flow-limiting degree maintains high, although the fluctuation of CT-FFR measurement values may be larger than other calcified lesions. The analysis by Tang et al., (9) also showed high CT-FFR performance in vessels with CAC ≥ 400. Kawaji et al. (11) evaluated the efficacy of CT-FFR in the real clinical application and noticed the comparable performance in vessels with extremely severe calcification (CAC ≥ 1,000) to those with CAC <1,000 (sensitivity: 100 vs. 91.3%, specificity: 62.5 vs. 50.0%). While, we noticed that all the sample sizes of vessels with severe calcification in previous investigations were small, which is consistent with the real-world situation. The impact of calcification severity on the discriminatory ability of CT-FFR in clinical use, especially CAC ≥ 400 or ≥ 1000, is still necessary to be explored.

It is worth mentioning that the difference of FFR and CT-FFR measurements was large and the discriminatory power of CT-FFR was relatively low in vessels of CAC = 0 group. In addition to the flow-limiting degree of lesions and cut-off value mentioned above, it can be else inferred that the identification of non-calcified plaques and vessel lumen by imaging segmentation algorithm is insufficient during the procedure of CT-FFR calculation. Although the AUC of CT-FFR in vessels with CAC = 0 (AUC: 0.84) is lower than calcified plaques, it was still consistent with the result from Nørgaard et al. (14).

There are some limitations in this study. First, the sample size of patients with severe coronary calcification is relatively small, although consistent with the real world. Second, CT-FFR was calculated based on computational fluid dynamics algorithm. It's still unknown whether the results can be generalized to the conditions of machine-learning CT-FFR calculation. A larger study population closely to the real clinical situation, especially those with CAC ≥ 400 or even ≥ 1,000, is necessary to confirm the results. This study provides scientific evidence of extensively integrating CT-FFR into clinical workflows and accurately guiding the clinical application of it.

## Conclusions

This study provides evidence that coronary calcification severity has no significant influence on the measurement and diagnostic performance of CT-FFR for identifying ischemia.

## Data Availability Statement

The raw data supporting the conclusions of this article will be made available by the authors, without undue reservation.

## Ethics Statement

The studies involving human participants were reviewed and approved by Ethics Committees of Fuwai Hospital, Beijing Chao-Yang Hospital, Sir Run Run Shaw Hospital, Qilu Hospital, and Teda International Cardiovascular Hospital. The patients/participants provided their written informed consent to participate in this study.

## Author Contributions

Clinical trial was managed by BL, BX, WC, HH, and FZ. Material preparation, data collection, and analysis were performed by NZ and YG. The manuscript was completed by NZ. All authors contributed to the study conception, design, collection, commented on previous versions of the manuscript, and participated the discussion.

## Funding

This study was supported by the Clinical and Translational Medicine Research Foundation of Chinese Academy of Medical Sciences (2019XK320065) and the Ministry of Science and Technology of China, National key research, and development project (2016YFC1300402).

## Conflict of Interest

The authors declare that the research was conducted in the absence of any commercial or financial relationships that could be construed as a potential conflict of interest.

## Publisher's Note

All claims expressed in this article are solely those of the authors and do not necessarily represent those of their affiliated organizations, or those of the publisher, the editors and the reviewers. Any product that may be evaluated in this article, or claim that may be made by its manufacturer, is not guaranteed or endorsed by the publisher.

## Abbreviations

FFR: fractional flow reserve
CAD: coronary artery disease
CCTA: coronary computed tomography angiography
CT-FFR: coronary computed tomography angiography derived fractional flow reserve
ICA: invasive coronary angiography
CAC: coronary artery calcification
PPV: positive predictive value
NPV: negative predictive value
ROC: receiver operating characteristics
AUC: area under receiver operating characteristics curve.
